# Development and Internal Validation of a Practical Model to Identify Observe Patients of the European Society of Cardiology 0/1-h Algorithm at Low Risk of a Coronary Diagnosis

**DOI:** 10.1159/000523718

**Published:** 2022-02-23

**Authors:** Murat Arslan, Eric Boersma, Admir Dedic, Eric A. Dubois

**Affiliations:** ^a^Department of Cardiology, Erasmus Medical Centre, Rotterdam, The Netherlands; ^b^Department of Radiology and Nuclear Medicine, Erasmus Medical Centre, Rotterdam, The Netherlands; ^c^Department of Intensive Care, Erasmus University Medical Centre, Rotterdam, The Netherlands

**Keywords:** Non-ST-elevation acute coronary syndrome, Acute chest pain, Observe group, Risk score

## Abstract

**Background:**

Patients with suspected non-ST-elevation acute coronary syndrome (NSTE-ACS) assigned to the “observe” zone of the European Society of Cardiology (ESC) 0/1-h algorithm form a heterogeneous group known to have an unfavourable prognosis. We aim to elucidate the clinical characteristics and management of these patients and generate a model that is predictive of a coronary diagnosis at index visit to the emergency department (ED).

**Methods:**

A retrospective observational cohort study, including adult patients presenting to the ED with suspected NSTE-ACS assigned to the “observe” zone of the ESC 0/1-h algorithm. Multivariable logistic regression analysis was performed for the prediction of a coronary diagnosis. Internal validation was performed using bootstrap resampling.

**Results:**

A total of 750 patients were included; mean age 66 ± 13 years, 35% women, 50% with prior history of coronary artery disease (CAD). In 372 (50%) patients a diagnosis was established within 30 days of index presentation, of whom 169 (45%) patients had a coronary-related event. Multivariable logistic regression analysis generated a 12-point risk score incorporating 5 variables for the prediction of such event, including type of angina, chest pain occurring during inspiration, prior history of CAD, ST-segment deviation on electrocardiogram, and estimated glomerular filtration rate <60. The final model had an optimism-corrected c-statistic of 0.78 (95% confidence interval [CI]: 0.74–0.82). A score <6 ruled out a coronary event in 276 (37%) patients, with a sensitivity and negative predictive value of 90% (95% CI: 84–94) and 94% (91–96), respectively.

**Conclusion:**

A score <6 identifies patients at low risk of a coronary diagnosis and can guide clinical decision-making in choosing the appropriate diagnostic test.

## Introduction

The introduction of high-sensitivity cardiac troponins (hs-cTn) has led to accelerated diagnostic protocols for the assessment of suspected non-ST-elevation acute coronary syndrome (NSTE-ACS) [1, 2, 3]. In the recent European Society of Cardiology (ESC) guideline on the management of patients with a suspected ACS without persistent ST-segment elevation, the use of a 0/1-h algorithm in centres with hs-cTn assays is recommended [4]. The ESC 0/1-h algorithm has two separate thresholds for “rule-out” and “rule-in” to optimize diagnostic accuracy and efficacy on both sides of the spectrum.

However, a substantial number of patients do not qualify for either category after repeated hs-cTn testing and are assigned to the “observe” zone. These patients are known to have an unfavourable prognosis, with 1-year survival rates comparable to patients assigned to the “rule-in” category [5, 6]. Management of these patients remains challenging, and hospital admission and additional (non)invasive testing are often necessary. A more individual approach is recommended where management decisions should be based on the degree of clinical suspicion of NSTE-ACS on a case by case basis [4]. The aim of this study was to elucidate the clinical characteristics and management of patients assigned to the “observe” zone and to generate a model that is predictive of a coronary diagnosis.

## Methods

This retrospective observational cohort study included 750 consecutive adult patients with suspected NSTE-ACS and acute chest pain as principal symptom. Patients presented to the emergency department (ED) at the Erasmus MC, University Medical Centre, Rotterdam, an academic tertiary referral hospital, between February 1, 2012, and January 31, 2020. Patients were included if they were aged 18 years and older and assigned to the “observe” zone of the ESC 0/1-h algorithm based on repeated hs-cTn measurements. The start date of February 1, 2012, was chosen as this corresponded with the introduction of the fifth-generation Elecsys hs-cTnT (hs-TnT) assay (Roche Diagnostics) in our centre. Although the 0/1-h algorithm was first recommended in the 2015 ESC guideline on the management of patients with a suspected ACS without persistent ST-segment elevation, we have also included patients that presented at the ED prior to this recommendation, with repeated hs-cTn measurements which would assign them to the “observe” zone based on current guidelines. Data regarding clinical characteristics, symptoms and signs, electrocardiogram (ECG) data, laboratory results, downstream testing, patient management, and diagnosis were obtained from electronic patient records. This study was conducted according to the principles of the Declaration of Helsinki and was approved by the local institutional review board.

### Diagnostic Procedure

Patients were categorized into three diagnostic groups if the diagnosis was established by treating physicians within 30 days of presentation to the ED: (1) coronary (including NSTE-ACS, chronic coronary syndromes, spontaneous coronary artery dissection, and coronary vasospasm); (2) cardiac, non-coronary (including non-ischemic cardiomyopathies, valvular heart disease, brady- and tachyarrhythmias, and [peri] myocarditis); (3) non-cardiac (containing all relevant non-cardiac diagnosis such as pulmonary embolism, anaemia, infections, malignancies, and malignant hypertension).

### Study Endpoint

The primary outcome was a coronary diagnosis at index visit to the ED, established by the treating physician, based on all available clinical data.

### Statistical Analysis

Continuous data are presented as mean ± SD or median (interquartile ranges), and categorical data are presented as proportions (percentages). We used logistic regression analysis for the prediction of a coronary diagnosis. Initially, univariable analysis was performed to evaluate the ability of chosen variables to predict a coronary diagnosis. We examined 29 potential predictors, including sex, age, cardiovascular risk factors, medication, symptoms, ECG findings, and laboratory results. Variables with a *p* < 0.10 entered the multivariate stage, and a multivariable logistic regression model was constructed to predict a coronary diagnosis, using the stepwise backward selection method, with a value of *p* = 0.05 as a model-entry criterion. Variables were checked for multicollinearity using correlations and variance inflation factors to avoid redundancy in the prediction model. Dichotomization of the continuous variable estimated glomerular filtration rate (eGFR) was based on the clinically relevant threshold for kidney disease. The relative magnitude of the model regression coefficients from statistically significant variables in the final multivariable model was used to calculate an individual patient's risk score for the prediction of a coronary diagnosis. Each point assigned to covariates in the final multivariable model was rounded to the nearest integer to simplify the calculation of the final score in clinical practice. The model discrimination abilities were evaluated by the c-statistic of the final multivariate model. For further internal validation, 1,000 bootstrap samples were obtained, and an optimism-corrected c-statistic was calculated [7]. The optimal cut-off value for the risk score was chosen after visual inspection of the diagnostic accuracy parameters at various cut-offs, with the aim to optimize the efficacy (defined as the number of patients ruled out) of the score while maintaining high sensitivity and negative predictive value (NPV) for the prediction of a coronary diagnosis. Receiver-operating characteristic curve analysis of the computed risk score was compared with a validated risk score, namely the History, ECG, Age, Risk factors, and Troponin (HEART) score [8, 9].

Survival data of all patients were acquired using in-hospital medical records and municipal civil registry. Cumulative survival of up to 1-year follow-up was calculated and stratified by diagnostic categories, i.e., (1) coronary; (2) cardiac, non-coronary, and (3) non-cardiac, with corresponding 95% confidence intervals and plotted using Kaplan-Meier curves. The log-rank test was used to examine differences between diagnostic categories in the Kaplan-Meier analyses, correcting for multiple comparisons using the Bonferroni method.

All statistical analyses were performed using SPSS version 24.0 (IBM, Armonk, NY, USA) and R version 4.0.2 (R Project for statistical computing, Vienna, Austria). All tests were two-tailed, and a *p* value <0.05 was considered statistically significant.

## Results

**Fig. 1:**
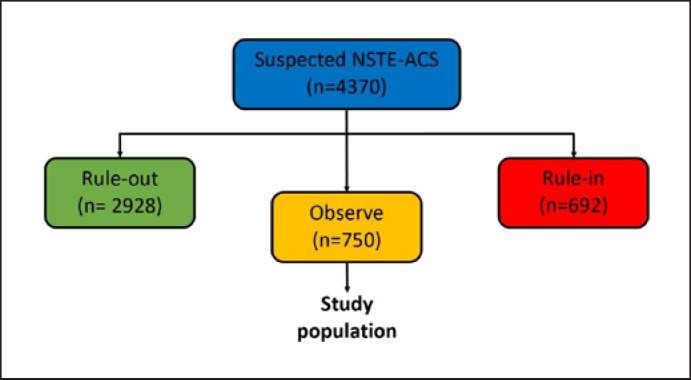
Flow chart of the study population. NSTE-ACS, non-ST-elevation acute coronary syndrome.

Between February 1, 2012, and January 31, 2020, 4,370 consecutive adult patients presented with suspected NSTE-ACS and acute chest pain, of whom 2,928 patients were triaged towards “rule-out” and 692 patients as “rule-in” by the ESC 0/1-h algorithm. In total, 750 suspected NSTE-ACS patients were assigned to the “observe zone” of the ESC 0/1-h algorithm and comprised the study population (Fig. 1). Baseline characteristics of included patients are shown in Table 1. The mean age was 66 ± 13 years and the proportion of women was 35%. Half of the patients had a prior history of coronary artery disease (CAD). In 32% of the patients first blood draw was performed within 3 h of chest pain onset. The median hs-TnT value in the study cohort at baseline was 18 (14–25) and the second median hs-TnT value was also 18 (14–25). Patients with a coronary diagnosis were more often male with pre-existing CAD and more often had ischemic ECG abnormalities as compared to the rest of the cohort (Table 1).

### Resource Use in the Observe Group

Non-invasive ischemia testing was performed in 54 (7%) patients within 30 days of index presentation and revealed signs of myocardial ischemia or infarction in 31 patients (Table 2). Invasive coronary angiography (ICA) was performed in 151 (20%) patients, of whom 92 (12%) patients underwent percutaneous coronary intervention and 3 (0.4%) patients underwent coronary artery bypass grafting within 30 days of index presentation. 263 (35%) patients were admitted to the hospital after evaluation at the ED and 411 (55%) patients were sent home with an appointment at the outpatient clinic (Table 2).

### Final Diagnosis

**Fig. 2:**
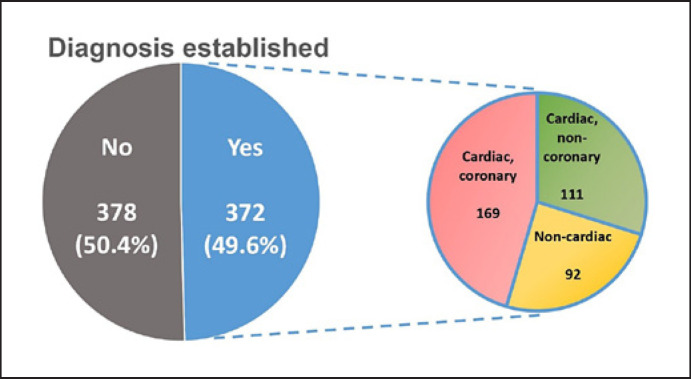
Figure depicting the proportion of patients with an established diagnosis related to index visit, based on all available clinical data with their corresponding diagnostic categories.

In 372 (50%) patients, a clinically relevant diagnosis related to index visit was established, based on all available clinical data (Fig. 2). The remaining 378 (50%) patients were discharged from the ED after exclusion of acute significant pathologies and had no obvious diagnosis after 30 days that explained their chest pain. Among the patients with an established diagnosis, the most common diagnosis was coronary disease, followed by “cardiac, non-coronary” diseases and finally non-cardiac diseases.

### Prediction of a Coronary Diagnosis

Univariable regression analysis of the relationship with a coronary diagnosis identified an association with female sex, hypertension, prior history of CAD, aspirin use, statin use, P2Y12 inhibitor use, calcium channel blocker use (a)typical angina, chest pain occurring during inspiration, heart rate, ST-segment deviation, and pathological Q waves on ECG and eGFR <60 (Table 3). Of these, prior history of CAD, (a)typical angina, chest pain occurring during inspiration, ST-segment deviation on ECG, and eGFR <60 remained significant independent predictors of a coronary diagnosis in the multivariable analysis. The final multivariate model had a *c*-statistic of 0.78 (0.74–0.82). Bootstrap internal validation revealed an optimism-corrected c-statistic of 0.77 (0.73–0.81).

**Fig. 3:**
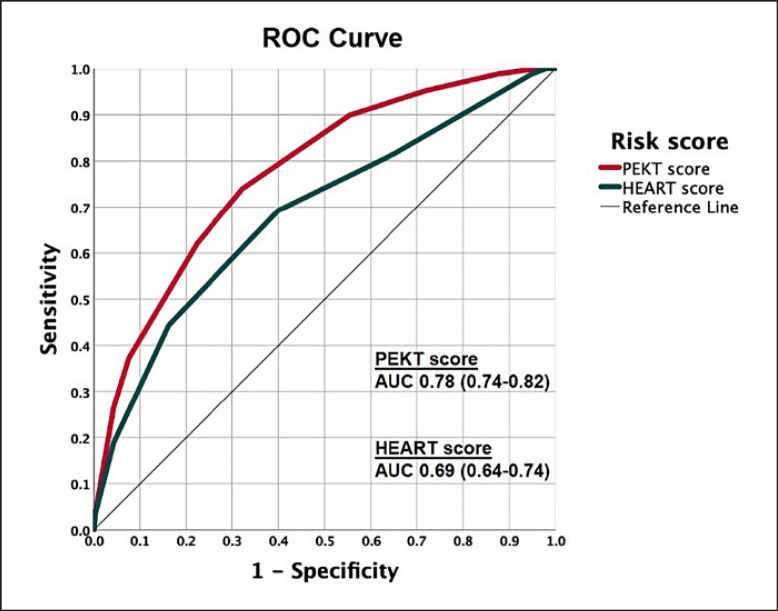
ROC curves of risk scores for the prediction of a coronary diagnosis. AUC, area under the curve; HEART score, history, ECG, age, risk factors, and troponin score; PEKT score, prior history, ECG, kidney function, and type of chest pain score; ROC, receiver-operating characteristic.

Points assigned to the five covariates in the final multivariable model are depicted in Table 4. From the final multivariable model, a 5-item risk score was generated with a score from 0 to 12. Prior history, ECG, kidney function, and type of chest pain (PEKT) score is composed of prior history of CAD (2 points), acute chest pain: atypical angina (2 points) or typical angina (4 points), chest pain occurring during inspiration (−3 points), ST-segment deviation (2 points), and eGFR <60 (−1 point), with all patients receiving an additional 4 points regardless of their symptoms at presentation. The median score in the study cohort was 6 [5, 6, 7, 8]. Sensitivity, specificity, positive predictive value, and NPV according to the PEKT score risk score are presented in Table 5. Bootstrap internal validation of the PEKT score revealed an optimism-corrected c-statistic of 0.78 (0.74–0.82). A PEKT score lower than 6 identified 276 (37%) patients at low risk of having a coronary diagnosis, with a sensitivity and NPV of 89.9 (84.4–94.0) and 93.8 (90.6–96.0), respectively. In comparison, the HEART score had a *c*-statistic of 0.69 (0.64–0.74) (Fig. 3).

### Survival

**Fig. 4:**
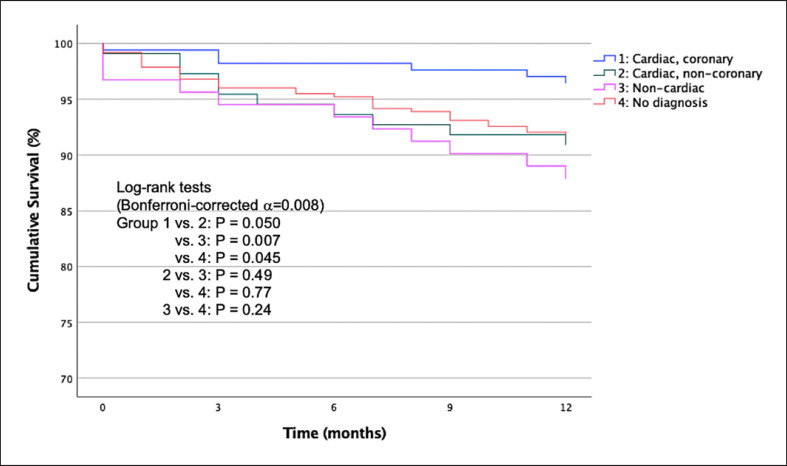
One-year Kaplan-Meier survival curves in patients assigned to the “observe zone” of the ESC 0 h/1 h algorithm stratified based on diagnostic categories.

The overall 30-day and 1-year cumulative survival in patients assigned to the “observe zone” were 0.98 (0.97–0.99) and 0.92 (0.90–0.94), respectively. The 30-day cumulative survival in patients with “no diagnosis,” “coronary,” “cardiac, non-coronary,” and “non-cardiac” diagnoses were 0.98 (0.97–0.99), 0.99 (0.98–1.00), 0.99 (0.97–1.00), and 0.97 (0.93–1.00), respectively. The 1-year cumulative survival in patients with “no diagnosis,” “coronary,” “cardiac, non-coronary,” and “non-cardiac” diagnoses were 0.92 (0.89–0.95), 0.96 (0.94–0.99), 0.91 (0.86–0.96), and 0.88 (0.81–0.95), respectively. Figure 4 shows the Kaplan-Meier plots stratified by diagnostic categories. Pairwise log-rank tests revealed a significant difference in 1-year cumulative survival between the cardiac, coronary group as compared to the non-cardiac group (*p* = 0.007, α = 0.008).

## Discussion

In this retrospective observational study, we investigated the clinical characteristics and management of suspected NSTE-ACS patients assigned to the “observe” zone of the ESC 0/1-h algorithm and we developed and internally validated the PEKT score, a simple 5-item tool to identify patients at low risk of a coronary diagnosis. We hereby report several important findings.

First, “observe” patients were generally elderly men with a prior history of CAD. Second, a clinically relevant diagnosis related to index visit at the ED was established in only 50%, with CAD being the most commonly established diagnosis. Third, cumulative 1-year survival in “observe” patients was 92% and patients with a coronary diagnosis appeared to have a relatively better survival outcome as compared to patients in other diagnostic categories.

Previous data from the Advantageous Predictors of Acute Coronary Syndromes Evaluation group portrayed similar clinical characteristics of “observe” patients as found in our study cohort, with the population generally being older men and with known pre-existing CAD [6, 12]. The proportion of men in our study was even higher in patients with a coronary-related diagnosis. Interestingly, in half of the patients, NSTE-ACS or a life-threatening condition was excluded, however, an underlying cause for their symptoms was not found within 30 days of presentation at the ED. Patients without a diagnosis still have an unfavourable prognosis beyond 30 days of presentation, representing a heterogeneous group of patients with complex (non-)cardiac causes of troponin leakage. Patients with a coronary diagnosis seem to have a better outcome when compared to other diagnostic categories. A possible explanation is that patients with a coronary diagnosis had a more readily treatable and simple event as compared to other diagnostic categories. In addition, we cannot rule out that patients without a coronary diagnosis may have had undiagnosed CAD, which untreated may have contributed to the survival differences.

### Resource Use

Our data shows that the treating physician had a preference for ICA as compared to other imaging modalities, such as coronary computed tomography angiography (CCTA), in patients assigned to the “observe zone.” The reason may be a combination of available expertise built over the years of using ICA and a lack of evidence for the usefulness of CCTA in the “observe zone.” Previous trials in low-risk patients with suspected NSTE-ACS have shown the efficacy of CCTA; however, this still remains to be seen for patients assigned to the observe zone [13, 14]. Currently, we are in the final phase of a prospective, double-blind, observational, multicentre study (COURSE trial) that aims to determine the usefulness of CCTA in patients with low-range positive troponins [15].

### PEKT Score

Patients in the “observe” group with pre-existing CAD have a more than 2-fold increase in the incidence of a coronary diagnosis when presenting to the ED with acute chest pain. Considering that in our study a large proportion of “observe” patients have pre-existing CAD, this is an important risk factor to take into consideration in further patient management decisions at the ED.

Acute chest pain plays a central role in our novel risk score, with typical angina having the greatest weight in predicting a coronary diagnosis among all 5 predictors. However, patients with chest pain which specifically worsened during inspiration have a clearly lower likelihood of having a coronary diagnosis. This highlights the importance of proper history taking in patients with chest pain complaints and its downstream effect on the decision to perform an ICA.

Patients in the “observe” group presenting with impaired renal function defined as eGFR <60 were less likely to have a coronary diagnosis as compared to patients with normal renal function. This phenomenon is partially ascribed to non-ischemic elevation of troponin levels in patients with kidney disease, as opposed to myocardial necrosis leading to a rise of troponin levels in patients with myocardial infarction [16, 17]. In patients with renal dysfunction, diagnostic protocols such as the ESC 0/1-h algorithm, also have a decreased specificity for type 1 myocardial infarction [18, 19, 20], i.e., even if patients with renal dysfunction develop myocardial infarction, it is less likely to have a coronary cause.

The PEKT score performed well and was able to discriminate between patients that need further coronary investigation and those that do not. A PEKT score lower than 6 identifies patients at low risk of coronary diagnosis with a relatively high NPV. In these patients treating physicians may consider an alternative diagnosis. It is important to note that our risk score warrants prospective external validation prior to use in daily clinical practice. The HEART score, which has been developed and validated in patients with suspected NSTE-ACS, was a relatively good predictor of a coronary diagnosis in our cohort but did not perform as well.

Our scoring system is simple and composed of easily obtainable clinical characteristics. The PEKT score may provide clinicians at the ED an opportunity to simplify their decision-making and may help reduce variability in patient management for the “observe” group.

### Limitations

The present study has several limitations that should be acknowledged. First, its single-centre, retrospective design has some inherent limitations, such as selection bias. Thus, the study sample may not be a true representation of “observe” patients, which limits generalizability. Second, the final diagnosis was established by treating physicians as opposed to a central adjudication process of final diagnoses. Lack of final diagnosis adjudication in turn may have affected the developed risk score, considering that a potential change in final diagnosis after adjudication would alter the outcome of our diagnosis-based risk score.

## Conclusion

Patients assigned to the “observe” group were in general elderly men with known pre-existing CAD. A substantial proportion of patients do not have an established diagnosis after 30 days and patients with a coronary diagnosis seem to have a better outcome when compared to other diagnostic categories. A PEKT score <6 identifies patients at low risk of a coronary diagnosis and may obviate the need for further coronary investigation in up to 40% of patients in our cohort.

## Statement of Ethics

This study was conducted according to the principles of the Declaration of Helsinki, approved by the Medical Research Ethics Committee of Erasmus Medical Center in Rotterdam, The Netherlands (registration number MEC-2020-0157) and a waiver of informed consent was obtained.

## Conflict of Interest Statement

The authors declare that there is no conflict of interest.

## Funding Sources

This work was supported by a grant from Erasmus MC and a research grant from the Erasmus MC Thorax Foundation (project grant B4).

## Author Contributions

Murat Arslan: conceptualization, data curation, formal analysis, project administration, visualization, and writing − original draft. Eric Boersma: formal analysis, methodology, investigation, and writing − review and editing. Admir Dedic: conceptualization, data curation, formal analysis, investigation, methodology, project administration, resources, supervision, visualization, writing − original draft, and writing − review and editing. Eric A. Dubois: conceptualization, data curation, formal analysis, funding acquisition, resources, supervision, visualization, writing − original draft, and writing − review and editing.

## Data Availability Statement

All data generated or analysed during this study are included in this article. Further enquiries can be directed to the corresponding author.

## Figures and Tables

**Table 1:** Baseline characteristics of patients assigned to the “observe zone” of the ESC 0/1-h algorithm

	Study cohort (*N* = 750)	Cardiac, coronary (*N* = 169)	Rest of the cohort (*N* = 581)	*p* value
Age, years	66±13	66±12	65±13	0.33
Female sex	259 (34.5)	43 (25.4)	216 (37.2)	0.005
Risk factors				
Active smoker	126 (16.8)	28 (16.6)	98 (16.9)	0.93
Hypertension	479 (63.9)	121 (71.6)	358 (62.0)	0.02
Dyslipidemia	448 (59.7)	117 (69.2)	331 (57.0)	0.004
Diabetes mellitus	227 (30.3)	59 (34.9)	168 (29.0)	0.14
Family history for CAD	188 (25.1)	53 (31.4)	135 (23.2)	0.03
PAD	65 (8.7)	11 (6.5)	54 (9.3)	0.28
Prior stroke/TIA	105 (14.0)	20 (11.8)	85 (14.6)	0.36
Prior history of CAD	375 (50.0)	120 (71.0)	255 (43.9)	<0.0001
Medication				
Aspirin	321 (42.8)	95 (56.2)	226 (38.9)	<0.0001
Oral anticoagulation	217 (36.1)	45 (26.6)	172 (29.6)	0.45
ACE inhibitor or ARB	416 (55.5)	98 (58.0)	318 (54.7)	0.45
P2Y12 inhibitor	164 (21.9)	46 (27.2)	118 (20.3)	0.06
Statin	449 (59.9)	115 (68.0)	334 (57.5)	0.02
Beta blocker	414 (55.2)	99 (58.5)	315 (54.2)	0.33
Diuretics	247 (32.9)	47 (27.8)	200 (0.34)	0.11
CCB	181 (24.1)	55 (32.5)	126 (21.7)	0.004
Symptoms and signs				
Chest pain*				
Typical angina	122 (16.3)	61 (36.1)	61 (10.5)	<0.0001
Atypical angina	346 (46.1)	82 (48.5)	264 (45.4)	
Non-anginal chest pain	282 (37.6)	26 (15.4)	256 (44.1)	
Chest pain occurring with inspiration	51 (6.8)	2 (1.2)	49 (8.4)	0.001
Blood pressure, mm Hg				
Systolic	145.5±42.0	145.7±23.2	144.1±26.4	0.48
Diastolic	82.2±15.3	83.2±14.8	82.1±15.0	0.40
ECG				
Sinus rhythm	622 (82.9)	147 (87.0)	475 (81.8)	0.11
Heart rate (per min)	75±18	73±14	77±20	0.03
Left ventricular hypertrophy	39 (5.2)	5 (3.0)	34 (5.9)	0.17
ST-segment deviation^#^	87 (11.6)	36 (21.3)	51 (8.8)	<0.0001
T Wave inversion	193 (25.7)	48 (28.4)	145 (25.0)	0.37
Pathological Q waves	79 (10.5)	26 (15.4)	53 (9.1)	0.02
Laboratory				
Time from chest pain onset to first blood draw				
<3 h	238 (31.7)	51 (30.2)	187 (32.2)	0.23
>3 h	337 (50.3)	71 (42.0)	266 (45.8)	
>24 h	155 (20.7)	44 (26.0)	111 (19.1)	
eGFR	69±22.8	72±21	68±23	0.04
Risk scores				
HEART score	5.3±1.6	6.1±1.6	5.1±1.5	0.0001
TIMI score	2.9±1.5	3.6±1.5	2.7±1.4	0.0001
GRACE score	118±29.7	119±29.3	118±29.8	0.66

Patients with ECGs suggestive of an ST-elevation myocardial infarction were excluded from this analysis. ACE, angiotensin-convertingenzyme; ARB, angiotensin II receptor blocker; CAD, coronary artery disease; CCB, calcium channel blocker; DOAC, direct oral anticoagulant; ECG, electrocardiogram; eGFR, estimated glomerular filtration rate; ESC, European Society of Cardiology; GRACE, The Global Registry of Acute Coronary Events; HEART, history; EKG, age, risk factors, and troponin; PAD, peripheral arterial disease; TIA, transient ischemic stroke; TIMI, the thrombolysis in myocardial infarction; VKA, vitamin K antagonist.

* Categorized according to the Diamond and Forrester classification [10].

# ST-segment deviation was defined as ≥1 mm ST-segment deviation in one or more leads [11].

**Table 2:** Use of diagnostic modalities and management of patients assigned to the “observe zone” of the ESC 0/1-h algorithm within 30 days of index presentation

(*N*on)-invasive diagnostic modalities used	Entire cohort (*N* = 750)	Various results per diagnostic modality [n = number of patients]
Non-invasive ischemia testing	54 (7.2)	Evidence of ischemia [22]
		Evidence of infarction [9] Non-diagnostic [4]
		Normal exam [19]

CT pulmonary embolism	35 (4.7)	Evidence of pulmonary embolism [3]

CT thorax angiography	18 (2.4)	Evidence of aortic dissection [1]

Coronary CTA	27 (3.6)	Normal or non-obstructive CAD (<50% stenosis) [18]
		1 vessel disease [2]
		2 vessel disease [4]
		3 vessel disease [3]

ICA	151 (20.1)	Normal or non-obstructive CAD (<50% stenosis) [38]
		1 vessel disease [43]
		2 vessel disease [28]
		vessel disease [42]

Management of patients		
Admission to hospital ward	263 (35.1)	−
Return to outpatient clinic	411 (54.8)	−
Revascularization		
PCI	92 (12.3)	−
CABG	3 (0.4)	−

Non-invasive ischemia testing included: Stress ECG, stress echocardiography; SPECT, and cardiac MRI, CABG, coronary artery bypass grafting; CAD, coronary artery disease; CCTA, coronary computed tomography angiography; ESC, European Society of Cardiology; ICA, invasive coronary angiography; PCI, percutaneous coronary intervention.

**Table 3:** Univariable analysis and adjusted multivariable analysis for the prediction of a coronary diagnosis

Covariate	Univariable analysis OR (95% CI)	*p* value	Multivariable analysis OR (95% CI)	*p* value
Female sex	0.58 (0.39–0.85)	0.005	−	−
Age (per 1-year increase)	1.007 (0.99–1.02)	0.32	−	−
Risk factors				
Smoking	0.94 (0.59–1.49)	0.80	−	−
Hypertension	1.54 (1.06–2.24)	0.023	−	
Dyslipidemia	1.32 (0.98–1.76)	0.067	−	
Diabetes mellitus	1.31 (0.91–1.89)	0.14	−	
Family history for CAD	1.42 (0.98–2.05)	0.064	−	
PAD	0.68 (0.35–1.33)	0.26	−	
Prior stroke/TIA	0.78 (0.47–1.32)	0.36	−	
Prior history of CAD	3.1 (2.16–4.54)	<0.0001	2.64 (1.75–3.98)	<0.0001
Medication				
Aspirin	2.02 (1.43–2.85)	<0.0001	−	−
Oral anticoagulation	0.86 (0.59–1.27)	0.45	−	−
ACE inhibitor or ARB	1.14 (0.81–1.61)	0.45	−	−
P2Y12 inhibitor	1.47 (0.99–2.18)	0.06	−	−
Statin	1.61 (1.11–2.31)	0.011	−	−
Beta blocker	1.21 (0.86–1.72)	0.28	−	−
Diuretics	0.73 (0.50–1.07)	0.11	−	−
CCB	1.74 (1.19–2.54)	0.004	−	−
Symptoms				
Chest pain*				
Non-anginal	1 (reference)	−	−	−
Atypical angina	3.06 (1.91–4.91)	<0.0001	3.06 (1.84–5.09)	<0.0001
Typical angina	9.85 (5.75–16.85)	<0.0001	8.55 (4.77–15.33)	<0.0001
Chest pain occurring during inspiration 0.130 (0.03–0.54)	0.005	0.18 (0.042–0.80)	0.02
ECG				
AF	1.49 (0.91–2.45)	0.11	−	−
Heart rate (per min)	0.99 (0.98–0.999)	0.03	−	−
Left ventricular hypertrophy	0.49 (0.19–1.27)	0.14	−	−
ST-segment deviation^#^	2.81 (1.76–4.49)	<0.0001	3.00 (1.75–5.14)	<0.0001
T Wave inversion	1.19 (0.81–1.75)	0.39	−	−
Pathological Q waves	1.80 (1.08–2.98)	0.023	−	−
Laboratory results				
eGFR <60	0.71 (0.49–1.03)	0.07	0.56 (0.37–0.86)	0.008

Patients with ECGs suggestive of an ST-elevation myocardial infarction were excluded from this analysis. ACE, angiotensin-converting-enzyme; AF, atrial fibrillation or atrial flutter; ARB, angiotensin II receptor blocker; CAD, coronary artery disease; CCB, calcium channel blocker; DOAC, direct oral anticoagulant; ECG, electrocardiogram; eGFR, estimated glomerular filtration rate; ESC, European Society of Cardiology; OR, odds ratio; PAD, peripheral arterial disease; TIA, transient ischemic stroke; VKA, vitamin K antagonist.

* Categorized according to the Diamond and Forrester classification [10].

# ST-segment deviation was defined as ≥1 mm ST-segment deviation in one or more leads [11].

**Table 4:** Individual variable scores of the multivariable model for the prediction of a coronary diagnosis

Variables	OR (95% CI)	Beta coefficient	Score
Prior history of CAD	2.65 (1.75–4.03)	0.969	2
Chest pain*			
Non-anginal	1 (reference)	−	0
Atypical angina	2.62 (1.58–4.37)	1.117	2
Typical angina	8.44 (4.69–15.17)	2.146	4
Chest pain occurring during inspiration	0.18 (0.042–0.79)	−1.702	−3
ST-segment deviation^#^	2.83 (1.64–4.87)	1.098	2
eGFR <60	0.61 (0.40–0.94)	−0.578	−1

In the final score, all patients receive an additional 4 points regardless of their symptoms at presentation. Patients with ECGs suggestive of an ST-elevation myocardial infarction were excluded from this analysis. CAD, coronary artery disease; CCB, calcium channel blocker; eGFR, estimated glomerular filtration rate; OR, odds ratio.

* Categorized according to the Diamond and Forrester classification [10].

# ST-segment deviation was defined as ≥1 mm ST-segment deviation in one or more leads [11].

**Table 5:** Predictive value of the “derived” risk score for coronary events at various cut-offs

Criterion	Criterion	Ruled out, *n* (%)	Sensitivity (95% CI)	Specificity (95% CI)	PPV (95% CI)	NPV (95% CI)
≥0	≥0	0 (0)	100.0 (97.8–100.0)	0.0 (0.0–0.6)	22.5 (22.5–22.5)	NA
>0	<1	3 (0.4)	100.0 (97.8–100.0)	0.5 (0.1–1.5)	22.6 (22.5–22.7)	100
>1	<2	14 (1.9)	100.0 (97.8–100.0)	2.4 (1.3–4.0)	23.0 (22.7–23.2)	100
>2	<3	24 (3.2)	100.0 (97.8–100.0)	4.1 (2.7–6.1)	23.3 (23.0–23.6)	100
>3	<4	75 (10.0)	98.8 (95.8–99.9)	12.6 (10.0–15.5)	24.7 (24.1–25.4)	97.3 (90.1–99.3)
>4	<5	170 (22.7)	95.3 (90.9–97.9)	27.9 (24.3–31.7)	27.8 (26.6–29.0)	95.3 (91.0–97.6)
>5	<6	276 (36.8)	89.9 (84.4–94.0)	44.6 (40.5–48.7)	32.1 (30.2–34.0)	93.8 (90.6–96.0)
>6	<7	438 (58.4)	74.0 (66.7–80.4)	67.8 (63.8–71.6)	40.1 (36.6–43.7)	90.0 (87.3–92.1)
>7	<8	515 (68.7)	62.1 (54.4–69.5)	77.6 (74.0–81.0)	44.7 (40.0–49.5)	87.6 (85.3–89.6)
>8	<9	643 (85.7)	37.3 (30.0–45.0)	92.4 (90.0–94.4)	58.9 (50.4–66.9)	83.5 (81.8–85.1)
>9	<10	680 (90.7)	26.6 (20.1–34.0)	95.7 (93.7–97.2)	64.3 (53.2–74.0)	81.8 (80.3–83.1)
>10	<11	739 (98.5)	4.7 (2.1–9.1)	99.5 (98.5–99.9)	72.7 (41.7–90.9)	78.2 (77.6–78.8)
>11	<12	743 (99.1)	3.6 (1.3–7.6)	99.8 (99.0–100.0)	85.7 (42.1–98.0)	78.1 (77.6–78.6)
»12	<13	750 (100)	0.0 (0.0–2.2)	100 (99.4–100.0)	NA	77.5 (77.5–77.5)

CI, confidence interval; NA, not applicable; NPV, negative predictive value; PPV, positive predictive value.
